# Flexural Strengthening of Reinforced Concrete Beams Using Near-Surface Mounted (NSM) Carbon Fiber-Reinforced Polymer (CFRP) Strips with Additional Anchorage

**DOI:** 10.3390/ma18112579

**Published:** 2025-05-31

**Authors:** Paweł Tworzewski, Kamil Bacharz

**Affiliations:** 1 Department of Building Structures, Kielce University of Technology, Al. Tysiąclecia Państwa Polskiego 7, 25-314 Kielce, Poland; 2 Department of Strength of Materials and Structures Diagnostics, Kielce University of Technology, Al. Tysiąclecia Państwa Polskiego 7, 25-314 Kielce, Poland; kbacharz@tu.kielce.pl

**Keywords:** debonding, fiber anchor, flexural strengthening, CFRP strip, concrete, near-surface mounted (NSM)

## Abstract

The work presents and examines a fiber anchoring system of NSM CFRP strips proposed for strengthening RC beams. The study included 11 beams: 3 unstrengthened beams, 3 beams strengthened with NSM CFRP strip without anchorage, and 5 beams strengthened with NSM CFRP strips with additional anchorage in two variants (the fiber anchor wrapped around the CFRP strip end and fan-folded on the beam surface; the fiber anchor connected with a 20 cm overlap to the strip). All beams were loaded until failure with two concentrated forces (four-point loading test). The measurements were carried out using digital image correlation (DIC). The obtained ultimate load values reached an average of 43.5 kN for unstrengthened beams, while for strengthened beams, they ranged between 56.6 kN and 60.2 kN. The strengthening efficiency was comparable for all beams regardless of the anchorage used and ranged from 29% to 37%. All strengthened beams failed due to strip debonding. The obtained results did not allow confirmation of the effectiveness of the proposed anchoring system. Detailed analysis showed that the lack of anchoring effectiveness was related to the debonding initiating factor, i.e., vertical crack opening displacement, which has not been described in proper detail by the researchers.

## 1. Introduction

Carbon fiber-reinforced polymers (CFRP) are a commonly used material in many sectors such as aviation, transportation, and construction. This is due to its unique properties, which include low weight, high strength, and corrosion resistance. When it comes to construction itself, the use of CFRP is constantly growing and is estimated at 6.2 kt in 2025 [1]. It should be emphasized here that 80 to 90% of CFRP use in the construction market is related to the strengthening and rehabilitation of existing structures due to their deterioration. It is worth mentioning that currently more and more attention is being paid to the development of carbon fiber-reinforced thermoplastic (CFRTP) composites and their structural engineering applications [2,3]. One of their most important properties is the possibility of recyclability.

The use of FRP materials to strengthen RC beams is the subject of many studies [4]. They are largely concerned with obtaining the best possible efficiency of this type of solution. The limitation of this type of solution is debonding, i.e., loss of the composite action. The occurrence of this type of failure leads in many cases to a reduction in the load-bearing capacity of the elements. It proceeds in two ways: normal stresses and high shear at the contact of the two materials lead to cracks in the FRP-concrete interface, which may also occur in the concrete cover due to other processes [5], ultimately leading to debonding of the FRP material or to separation of the concrete cover together with the FRP material, initiated by a crack near the end of the FRP [6,7,8,9,10,11,12,13]. Debonding and related failure can occur between different interfaces. Debonding in the concrete most often occurs because its tensile and shear strength is lower than that of the epoxy resin. Debonding in the adhesive rarely occurs because the situation must be the opposite, i.e., the strength of the epoxy resin must be lower than that of the surrounding concrete. Debonding at the interfaces between concrete and adhesive or adhesive and FRP also rarely occurs because it is usually associated with insufficient preparation of the material surface. Debonding inside the FRP occurs between the fibers and the resin in the strip itself and occurs in the case of high concrete strength. This behavior should be taken into account when estimating the load-bearing capacity of RC beams strengthened using FRP materials. This topic is discussed in many works [14,15,16,17,18,19]. The use of additional anchorage can successfully change the failure mode from FRP debonding to steel yield followed by rupture of the FRP or steel yielding followed by crushing of compressive concrete without rupture of the FRP [20,21,22].

The development of an efficient anchor system is one of the most significant challenges when it comes to the use of CFRP materials for strengthening RC beams. A certain difficulty is the properties of CFRP itself, i.e., anisotropic properties: high tensile strength with low compressive and shear strength [23].

Studies related to the use of different types of mechanical anchoring systems for the flexural strengthening of reinforced concrete beams are presented in many works [20,24,25,26,27,28]. They confirm that their use allows for higher load-bearing capacity of the elements and reduction of ductility. This applies to both the EBR (externally bonded reinforcement) and NSM (near-surface mounted) systems. The results obtained (increased in the range of 17% to 50%) are, of course, diversified depending on many factors, including reinforcement ratios [29]. The authors presented a detailed description of various anchoring systems in [30,31]. In the case of EBR systems, the debonding process is quite well recognized, and there are many analytical models available in the literature to predict debonding failure, as well as solutions to limit this phenomenon. As for NSM systems, as this is a newer solution, there are few analytical models and solutions in the field of anchoring [10,11,32,33,34].

Metallic anchors using steel plates and bolts for CFRP plates are one of the quite popular solutions used in the case of anchoring in EBR systems. They are most popular in the case of strengthening large objects, i.e., for bridge or cable structures. The following solutions can be distinguished: mechanical anchor, adhesive bonding anchor, and hybrid anchor. These systems are constantly being developed in order to reduce problems resulting from the susceptibility of metal elements to environmental impact, stress loss in the CFRP, the occurrence of local damage during gripping, and optimization [23,35,36]. The use of this type of solution allows for the application of prestressing and thus a significant increase in the efficiency of CFRP use.

One of the well-known nonmetallic anchor systems for strips and sheets is the use of FRP U-jackets [20,22,24,25,28]. This is an effective solution for EBR-type reinforcements. Additionally, it can be used simultaneously as a flexural and shear strengthening of RC beams. Studies of this type of solution indicate that under appropriate conditions, plate end debonding can be completely eliminated. In the case of NSM-type reinforcements, their effectiveness is limited [24].

**Figure 1:**
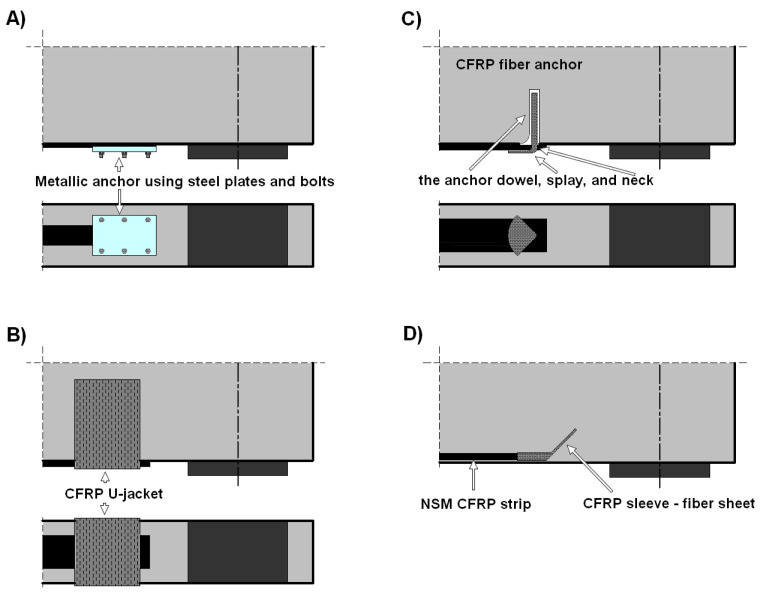
Examples of anchoring systems: (**A**) steel plates and bolts; (**B**) CFRP U-jacket; (**C**) fiber anchor; (**D**) fiber sheet anchor.

Another solution is the use of composite anchors made of fiber cords [21,26,27,29,31,37,38]. Another term for this type of solution is spike anchor. They are adhesively bonded into holes made in the beam web, whereas the rest outside the hole is fan-folded on the strip or sheet surface. Based on this, in the construction of this type of solution, three parts can be distinguished (Figure 1C): the anchor dowel, splay, and neck. The anchor dowel is the part of the anchor adhesively bonded in the pre-drilled hole. The anchor splay is the part fan-folded on the strip or sheet surface. Both parts are connected by a neck. The shape of the fan (fan length, bonding area, fan angle) is important because it is designed to minimize stress concentrations along the length of the spike anchor [39]. Previous research has shown that the most important parameters affecting the effectiveness of this solution include: anchor size and pre-drilled hole size, anchor dowel length, the shape of the anchor fan, bend radius, and bond condition [39,40,41]. The following guidelines are given in [40] for this type of solution: the recommended fan angle is 45°; the minimum embedment length (anchor dowel length) is 102 mm; the recommended anchor bend radius is 13 mm; the ratio of the hole area to the cross-sectional area of the CFRP anchor is expected to be 2.2 and should be no greater than 4.8; the minimum dimensions recommended for the CFRP patches are equal to the strip width in both directions, and the patches are suggested to extend 38 mm beyond the center of the anchor hole; a minimum of 50 mm of bond length is recommended to apply in front of the anchor fan; a good bond condition is required. In [39], it was observed that the use of a longer and narrower anchor fan (tested variants of fan angle/anchor fan length 30°/85 mm, 45°/120 mm, 60°/180 mm) allows for obtaining higher load-bearing capacities of the tested samples. Spike anchors can be used in many places along the length of the beam. However, a large number of anchors increases installation time and costs. For example, in [27], elements with additional composite anchors showed a 30% increase in load capacity.

A rather interesting solution, similar to fiber anchors, is presented in [24]. The anchor itself is made of a fiber sheet and is used only at the ends of the strip. Unlike previous solutions, this one is dedicated to NSM-type reinforcements. The research results show that the use of this solution led to an increase in the load-bearing capacity of the NSM-strengthened beam by 13% to 35% and was more effective than the use of FRP U-jackets.

**Figure 2:**
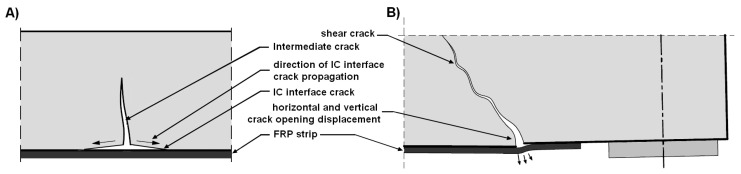
Debonding caused by (**A**) intermediate crack debonding (IC debonding) and (**B**) horizontal and vertical crack opening displacement in a shear crack.

However, it should be emphasized that anchoring methods allow for the elimination or delay of debonding, but not always. Intermediate crack debonding (IC debonding) (Figure 2A) is a very dangerous type of failure because there is no way to prevent it. Usually, this failure is the result of horizontal crack opening displacement, but it can also be the result of vertical crack opening displacement [15,34]. The place where the crack occurred in this case is the place where debonding is initiated, but failure occurs only when the interfacial shear stress reaches the maximum shear resistance (Figure 2B).

New and existing anchorage systems for FRP materials used for strengthening RC beams still require attention. In this article, the authors present the research results of the proposed anchoring system concept using a fiber composite anchor for NSM-type strengthening. At the same time, a specific failure of the tested beams was observed as a result of debonding caused by vertical crack opening displacement, which has not been quantified in proper detail yet.

This concept of the proposed anchoring system is the result of the research presented by the authors in [31], where a fiber anchor (CFRP rope) was used for flexural strengthening of RC beams, and the concept presented in [24,42]. The CFRP anchor was adhesively bonded to a hole drilled perpendicularly to the beam web at the end of the strip. Cooperation between the strip and the anchor was ensured by a 20 cm overlap connection. The described concept is shown in Figure 1. This solution is easy to apply and can also be used successfully as a beam shear reinforcement. This requires gluing the anchor into a hole made through the entire height of the beam web.

## 2. Materials and Methods

### 2.1. Materials Used to Prepare Specimens and Their Properties

**Figure 3:**
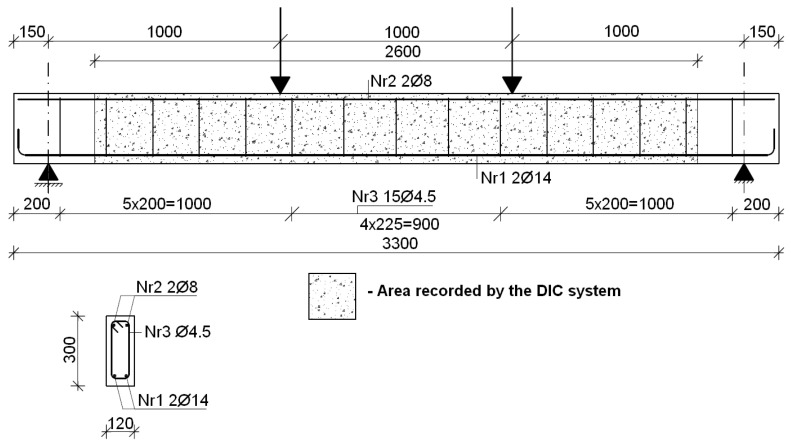
Reinforcement scheme of test beams.

The results concern the tests of 11 single-span reinforced concrete beams with a total length of 3.30 m and a rectangular cross-section of 0.30 m × 0.12 m. As beam reinforcement, two Ø14 rebars were used as bottom reinforcement and two Ø8 rebars as top reinforcement, all made of B500SP steel. The manufacturing drawing of the beam reinforcement is shown in Figure 3. The reinforcement ratio of the steel bars was ρ_s_ = 0.93%. Rebar tensile testing was performed on 42 samples, and the results are presented in Table 1. During the concrete casting, cube-shaped samples were taken: 150 mm × 150 mm × 150 mm. All beams were cast at a similar time (2017, precast factory: Sibet S. A., Kielce, Polska) in several-day intervals due to the precast form allowing for the production of 4 elements/beams at one time. The strength test of the concrete samples was carried out in parallel to the beam tests. The beams BW5-11-M1 and BW5-11-M2 were tested in 2024, while the remaining ones were tested in 2018. Therefore, the concrete strength results are given separately. Two concrete mixtures of different strength classes were used to prepare the beams:

- beams marked with the symbol 11 (BN-11-M1, BW3-11-M1, BW4-11-M1)—concrete average cylinder compressive strength *f_c_* = 50.1 MPa (12 samples).
- Beams with the symbol 12 (BN-12-M1, BW3-11-M1, BW3-11-M2, BW4-11-M1, BW4-11-M2)—concrete average cylinder compressive strength *f_c_* = 57.1 MPa (36 samples) and (BW5-11-M1, BW5-11-M2)—concrete average cylinder compressive strength *f_c_* = 53.5 MPa (24 samples).

### 2.2. Strengthening of the Specimens

**Figure 4:**
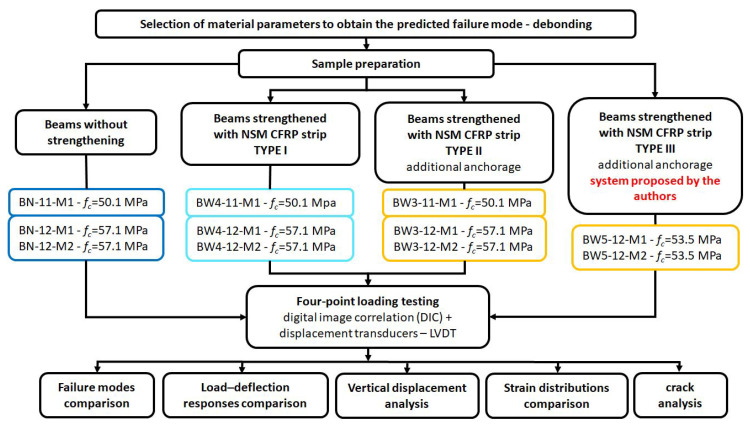
Flowchart of the research methodology.

The CFRP strip was used as flexural strengthening in each RC beam, obtaining a composite strengthening ratio of ρ_f_ = 0.16%. Differences are related to the use of additional anchoring and its method. The beams are divided into four types (Figure 4):

- Reference beams unreinforced BN—3 elements;
- BW4 beams—3 elements. RC beams strengthened using the NSMR (near-surface mounted reinforcement) without additional anchoring;
- BW3 beams—3 elements. RC beams strengthened using the NSMR method with additional anchoring in the near-support zone at the end of the strip—the fiber anchor wrapped around the CFRP strip end and fan-folded on the beam surface;
- BW5 beams—3 elements. RC beams strengthened using the NSMR method with additional anchoring in the support zone at the end of the strip—the fiber anchor connected to the strip with a 20 cm overlap.

The first stage of preparation of the strengthened specimens was the same for each beam. It required several steps:

1. Cutting the grooves using a wall chaser with dust extraction (the groove dimensions 10 mm × 20 mm were adopted in accordance with the manufacturer’s requirements [43,44]);
2. Cleaning the grooves and FRP surfaces.

**Figure 5:**
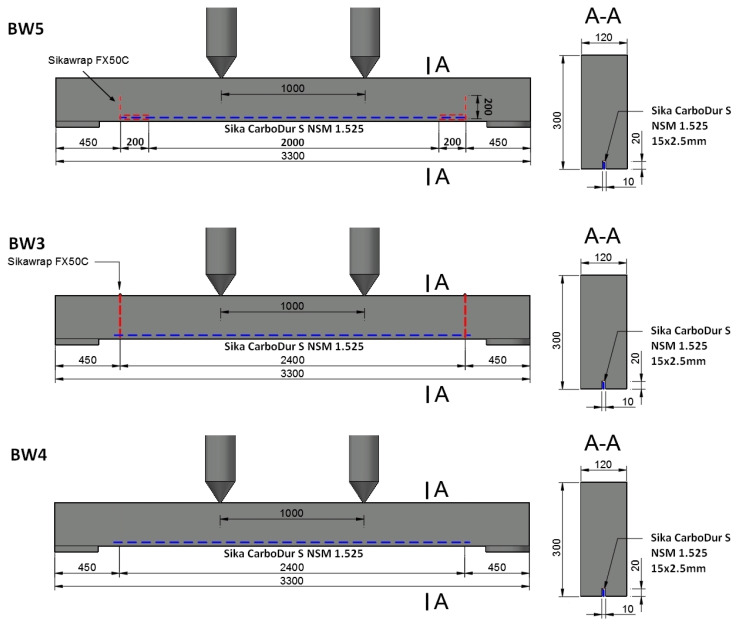
Schemes for strengthening RC beams.

**Figure 6:**
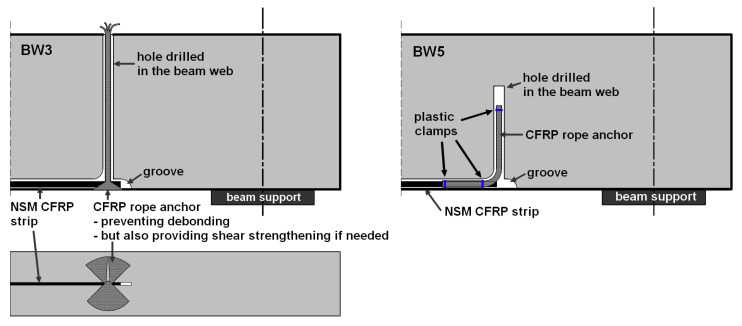
CFRP strip anchorage details.

**Figure 7:**
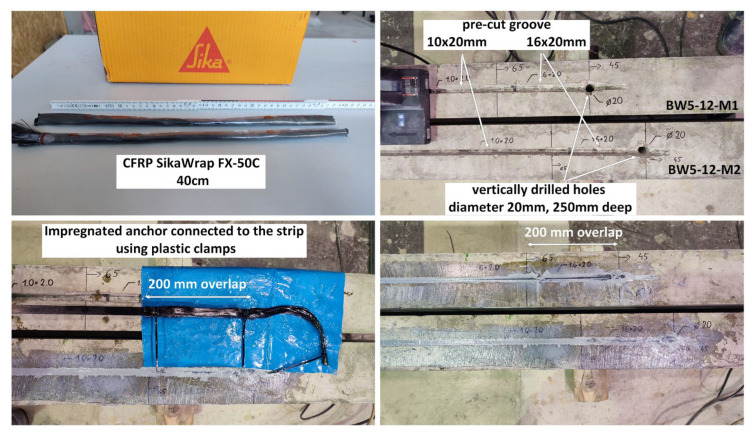
Installation steps for NSM strip with additional anchorage—BW5 beams.

The remaining stages varied between beams:

- BW4 beams—strengthening followed Figure 5:

1. Filling pre-cut groove with Sikadur-330 resin [43];
2. Gluing Sika CarboDur S NSM 1.525 CFRP strip (dimensions of the tape cross-section: 15 mm × 2.5 mm);
3. Refilling or removing excess resin.

- BW3 beams—strengthening followed Figure 5 and Figure 6:

1. Drilling vertical holes (20 mm in diameter) in the beam web 30 cm from the support axis, through the entire height of the beam cross-section;
2. Filling pre-cut groove and drilled hole with Sikadur-330 resin;
3. Impregnating SikaWrap FX-50C Sika CFRP anchors along their entire length with Sikadur-52 resin [44];
4. Gluing the fiber anchor into the drilled holes leaving a 6 cm section outside the hole;
5. Dividing the protruding part of the fiber anchor into two parts;
6. Gluing the CarboDur S NSM 1.525 CFRP strip in the pre-cut groove;
7. Wrapping the strip end on both sides with the part of the anchor left outside the hole to obtain a 5 cm overlap;
8. Spreading the fiber anchor on the beam surface;
9. Refilling or removing excess resin.

- BW5 beams—strengthening followed Figure 5, Figure 6 and Figure 7:

1. Widening the groove (dimensions of the widened groove: 16 mm × 20 mm) in the section of the planned strip and fiber anchor connection;
2. Drilling vertical holes (20 mm in diameter; hole depth: 25 cm [45]) in the beam web 30 cm from the support axis;
3. Filling pre-cut groove and drilled hole with Sikadur-330 resin;
4. Impregnating SikaWrap FX-50C Sika CFRP anchors along their entire length with Sikadur-52 resin;
5. Connecting the anchor with an overlap (20 cm) with CarboDur S NSM 1.525 CFRP strip using plastic clamps;
6. Gluing the strip into the pre-cut groove and the anchor into the drilled hole using a wire hooked to a plastic clamp installed at the end of the anchor fibers;
7. Refilling or removing excess resin.
8. In order to make pre-cut grooves and holes, a wall chaser (Bosch GNF 35CA) and a hammer drill were used. The adopted method of preparing beams for gluing the composite from the point of view of practical application does not differ in the degree of complexity from other construction works.

**Figure 8:**
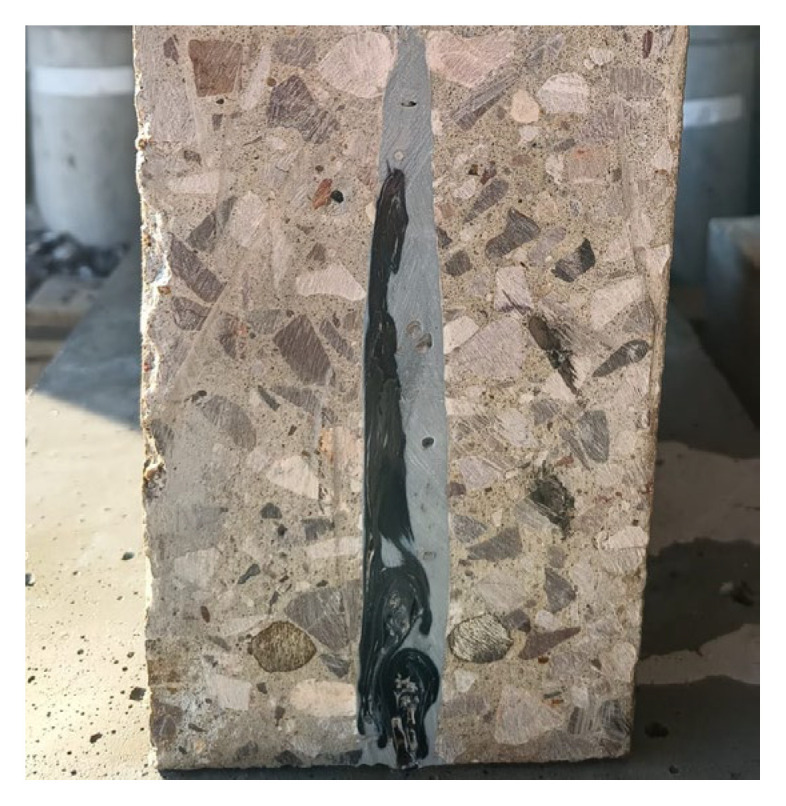
Vertical cross-section through the anchor point—BW5-12-M1 beam.

Photos of subsequent stages of strengthening RC beam BW5 are shown in Figure 7. The parameters of the materials used to prepare the beams are presented in Table 1. A cross-section of the beam with a visible anchor, made using a circular saw to check the workmanship quality, is shown in Figure 8—visible small empty spaces not filled with resin were left after removing the steel rod used to press the fiber anchor into the hole filled with resin.

The anchoring solution used in the BW3 beams was based on the solutions used in the EBR systems, with the difference resulting from the direction of the anchor fan distribution, which was aimed at obtaining the greatest possible contact between the CFRP strip and the anchor fibers.

All beams were loaded monotonically to failure—force-controlled four-point loading (constant speed 0.4 kN/min) using two actuators (maximum force 400 kN) set at a spacing of 1 m. The axial spacing of the test stand supports was 3 m. Spherical bridge bearing supports were used. A photo of the test stand and supports is shown in Figure 7. The measurements were carried out using the following:

- digital image correlation (DIC)—to measure deflections at selected points, strains, location, and width of cracks on the beam surface observed by cameras;
- five displacement transducers, LVDTs, used for measuring beam deflections;
- the moment of failure of each beam was recorded by a camera placed on a tripod on the other side of the beam relative to the one recorded by the DIC system.

**Figure 9:**
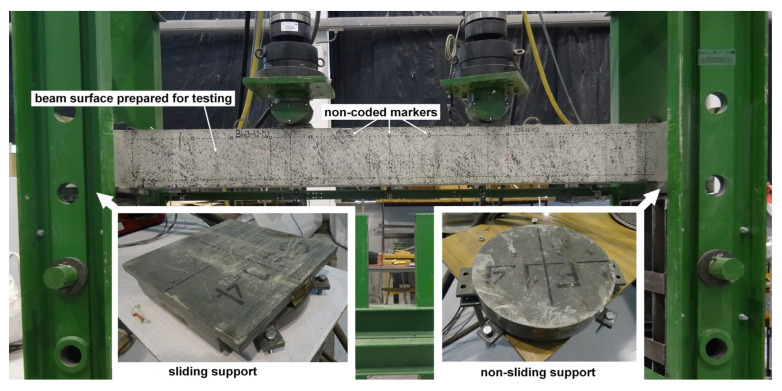
Test setup used in testing.

Digital image correlation (DIC) is a versatile tool that provides a wide range of measurement possibilities [46,47,48,49,50]. Tracking the deformation of an element is possible thanks to the recording of its suitably prepared surface using cameras. In the case of reinforced concrete beams, it is sufficient to apply a black pattern to its surface (Figure 9). In the case of BN, BW3, and BW4 beams, the ARAMIS 5M system (Braunschweig, Germany) and two sensors (camera spacing 1200 mm) equipped with monochrome Baumer TXG50 cameras (Frauenfeld, Switzerland) with a resolution of 2448 × 2050 pixels were used for the measurements. In the case of BW5 beams, the newer ARAMIS SRX system and one sensor (camera spacing 1200) equipped with cameras with a much higher resolution of 4096 × 3068 pixels were used.

Noncoded markers were adhesively bonded to the surface of the beams at 20 cm intervals at the height of the center of gravity of the compressed and tensioned reinforcing bars (Figure 9).

## 3. Results

### 3.1. Failure Modes

**Figure 10:**
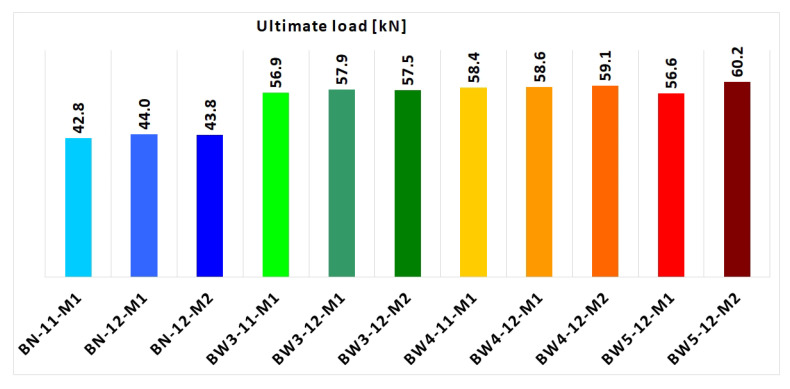
Experimental load-bearing capacity of all RC beams.

To estimate the predicted load-bearing capacity of the beams taking into account the debonding of the FRP, the strain in the FRP reinforcement was limited to the strain 0.7*ε_fu_* (where *ε_fu_*—laminate elongation at break in tension). The load-bearing capacity values obtained for each beam are presented in Table 2 and Figure 10.

BW4

**Figure 11:**
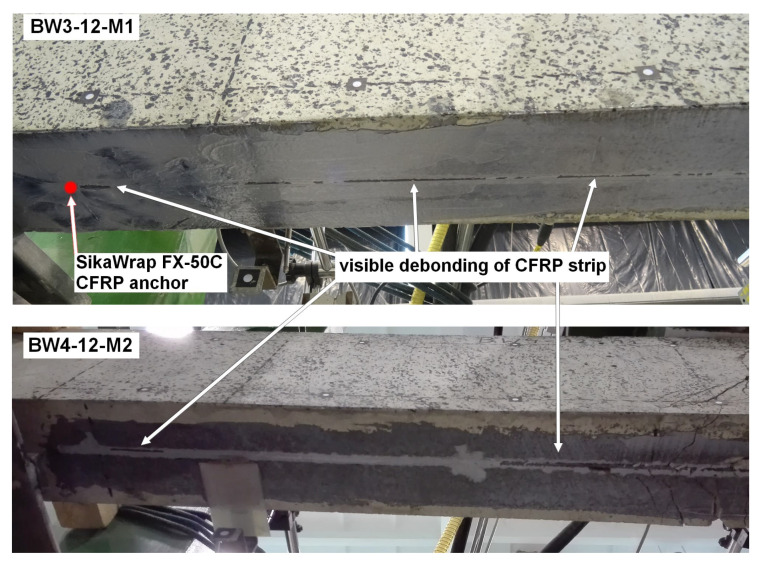
Failure mode of beams BW3-12-M1 and BW4-12-M2.

The description of failure modes began with the BW4-type beams because they were the only strengthened RC beams that did not have an additional anchorage of the CFRP strip at its end. All specimens failed in a similar manner, i.e., debonding of the CFRP strip initiated next to the sliding support leading to the loss of adhesion of the strip to the resin along its entire length (Figure 11). The experimental load-bearing capacity for each of the tested RC beams was lower than the predicted value, on average by approximately 1.46% (Table 2).

BW3

In the case of all BW3 beams, the additional anchorage solution applied did not prevent the strip from debonding. Apart from the BW3-11-M1 beam, in which the adhesion of the strip to the resin was intact in the anchorage area on the left side, the strip debonding in other beams covered the entire length and proceeded in the same way as in the case of the BW4 beams (Figure 11). The experimental load-bearing capacity of the BW3 beams was lower than the value predicted from the calculations, on average by approximately 3.53% (Table 2). On the other hand, the experimental load-bearing capacity was lower than that in the case of the BW4 beams, despite the additional anchorage by an average of 2%. Considering the small contact area between the CFRP strip and the anchor, this type of solution can be considered insufficient for NSM-type strengthening.

BW5

**Figure 12:**
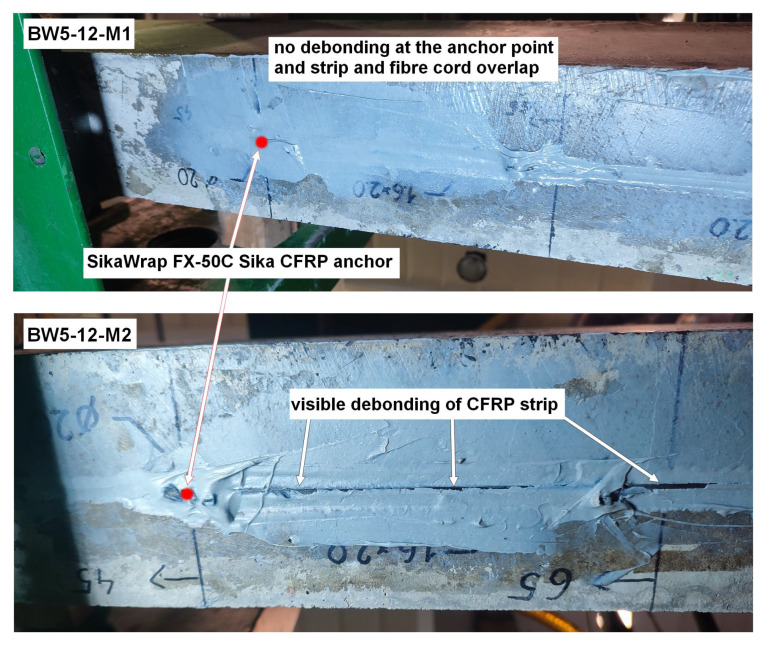
Failure mode of beams BW5-12-M1 and BW5-12-M2.

In the case of BW5 beams, both elements differ from each other in both failure mode and load-bearing capacity. In the case of the BW5-12-M1 beam, no debonding was observed on the fiber anchor overlap length on both the left and right sides of the beam, but it occurred on the remaining part of the strip (Figure 12). Although the anchorage fulfilled its function, the value of the load-bearing capacity was the lowest among all reinforced RC beams. The opposite situation was observed in the BW5-12-M2 beam, in which the debonding of the CFRP strip covered its entire length. The strip lost adhesion on the length of the overlap with the CFRP anchor (Figure 12) and the obtained load-bearing capacity had the highest value among all tested beams, higher than the BW5-12-M1 beam by approximately 6%. Only in the case of the BW5-12-M2 beam did the experimental load-bearing capacity slightly exceed the predicted value by 0.7%.

Close inspection revealed that debonding inside the CFRP strip had occurred—fibers from the outer surface of the strip remained attached to the resin after the beam failure. This is due to the relatively high strength of the RC beams.

### 3.2. Load–Deflection Responses

**Figure 13:**
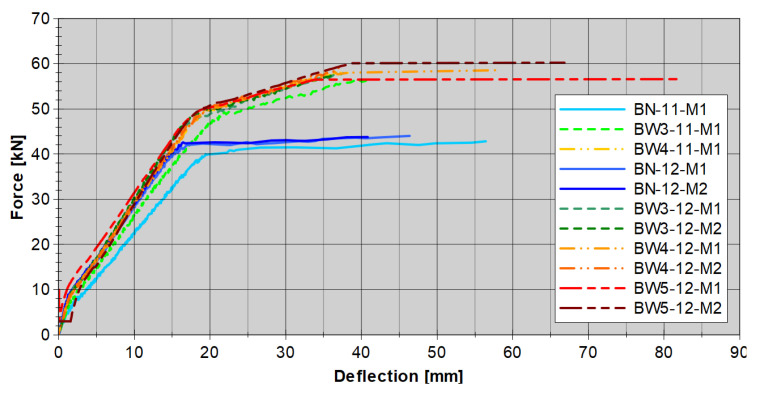
Loads versus midspan deflection for tested reinforced concrete beams.

An important point in the analysis of the behavior of strengthened RC beams is the load–deflection relationship. The measurement results are shown in Figure 13. Based on the results obtained, it can be stated that the load-deflection responses for all reinforced RC beams are similar. A faster increase in deflection can be observed in the case of beams made of lower-class concrete BN-11-M1 and BW3-11-M1. The BW5-12-M2 beam, whose CFRP strip was additionally anchored with composite anchors connected to the strip itself with a 20 cm overlap (Figure 7), had the highest stiffness and the highest load-bearing capacity. The indicated stiffening had only a local character resulting from the increased cross-section caused by the overlap.

The deflection course of the elements during the loading process is also worth noting. In the attached graphs, two turning points can be seen on each graph. The first occurs at a load of about 5 kN for each of the forces and is related to the cracking of the concrete in the tension zone. The second turning point, at a level of about 40 kN for unstrengthened beams and 50 kN for strengthened RC beams, is related to the yielding of the tension steel bars. In the first phase (up to the second turning point), the beams are characterized by very similar behavior. Only the BN-11-M1 beam shows increased deflection. The fundamental change occurs after the second turning point, where the steel plasticizes and failure occurs in the unstrengthened RC beams. In the case of strengthened RC beams, the slope of the load-deflection relationship changes and deflection increases faster, and the failure itself occurs much later.

At the last phase of the load, we can see a flattening in the graph (Figure 13). It is related to the adopted method of load control. In the conducted tests, this control was carried out by setting a constant value of the force increase, at the level of 0.4 kN/min. Hence, when the element approaches failure and debonding of the CFRP strip appears, the apparatus accelerates the actuator’s motion to achieve the desired increase in force. This results in a significant increase in deformation without an increase in force, because the element’s response is too small for the load level to increase. At the same time, no significant effect of anchoring was observed, aside from the slightly greater stiffness of the BW5-type beams.

### 3.3. Analysis of Vertical Displacements

**Figure 14:**
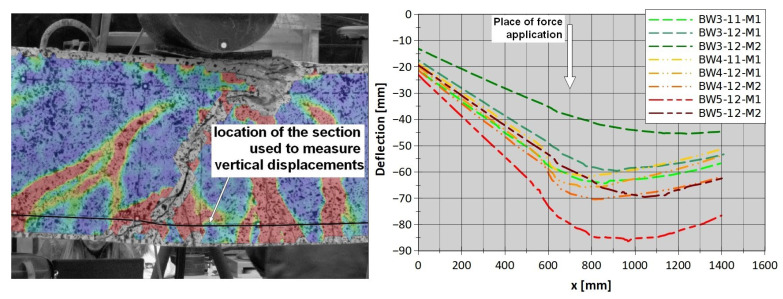
Vertical displacement measurement results for the beams at the stage recorded just after the debonding of the CFRP strip.

To understand why strip debonding occurred in all strengthened RC beams, regardless of the anchorage used, vertical displacements were analyzed in detail for the horizontal section created along the tension reinforcement (Figure 14). The course of vertical displacements along the length of each beam on its right side (near the sliding support) at the stage just after strip debonding was observed is shown in Figure 14. For each of the beams, a curvature can be observed in the area under the concentrated force. This is related to the vertical displacement at the crack location, which allows us to conclude that the vertical crack opening displacement was the initiating cause of the debonding.

### 3.4. Strain Distributions

**Figure 15:**
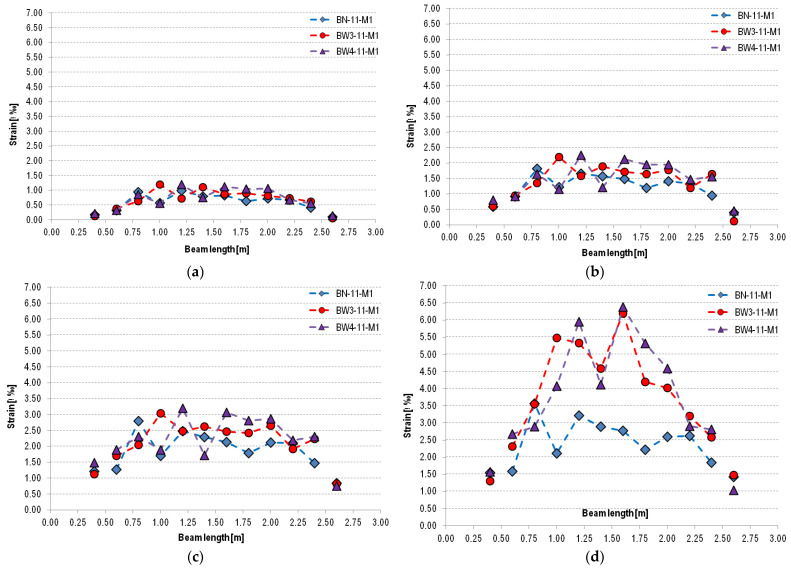
Strain distribution on the level of the tensile reinforcement on selected levels: (**a**) 30%, (**b**) 50%, (**c**) 70%, and (**d**) 90% of the ultimate load for beams of type 11.

**Figure 16:**
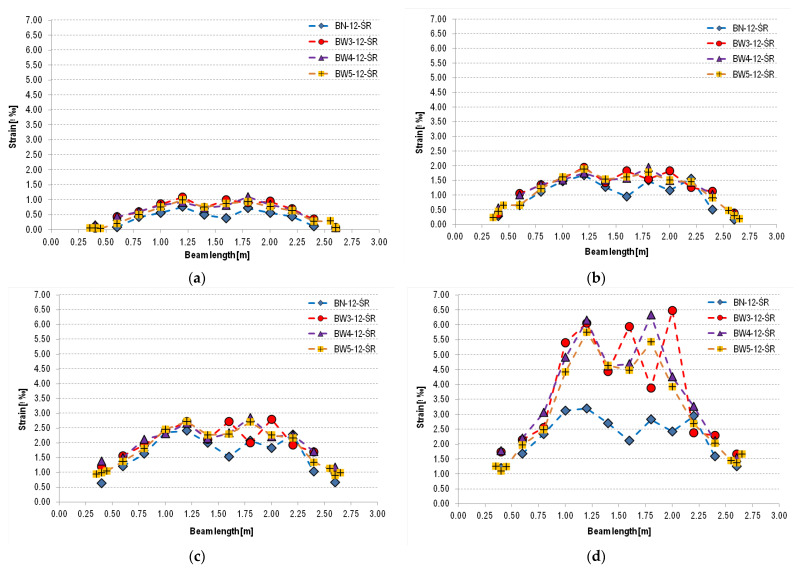
Strain distribution on the level of the tensile reinforcement on selected levels: (**a**) 30%, (**b**) 50%, (**c**) 70%, and (**d**) 90% of the ultimate load for beams of type 12.

**Figure 17:**
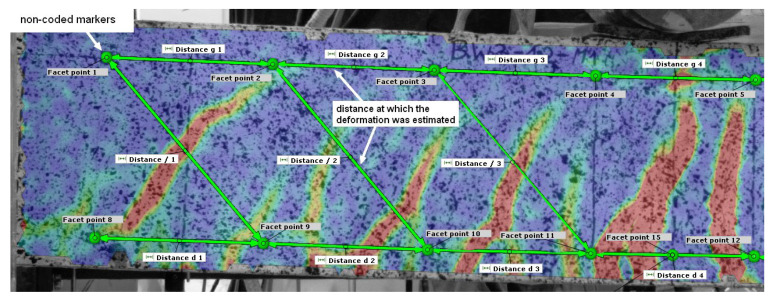
Location of points/non-coded markers and sections used to determine strain values.

Similar to the work presented in [30], a strain distribution analysis of reinforced concrete beams was performed. Strain measurement was performed at the height of the center of gravity of the tensile reinforcement (Figure 15 and Figure 16). The arrangement of non-coded markers and the measuring sections created between them is shown in Figure 17.

On this basis, it can be stated that at the lowest load level (30% of the ultimate load for a given type of beam), type 11 beams (BN-11-M1, BW3-11-M1, BW4-11-M1) show similar deformations. In the case of type 12 beams (BW3-12-ŚR, BW4-12-ŚR, BW5-12-ŚR), increased deformation can be observed near the center of the beams compared to unstrengthened elements (BN-12-ŚR). A similar relationship can also be observed for loads at 50% and 70% of the ultimate load. The greatest differences in strains measured at the center of gravity of the tensile reinforcement can be observed at a load level of 90% of the ultimate load. At this level of element load in both groups of beams, i.e., 11 and 12, it can be observed that the strains of reinforced beams are significantly greater than those of unreinforced beams. Reinforced beams of group 11 (BW3 and BW4) show a similar level of strain, with a maximum of about 6.5‰, which is almost twice as much as in the case of unreinforced beams, where the maximum strain is about 3.25‰. This is because strengthened RC beams are able to transfer greater loads and have reduced ductility. In the case of type 12 beams, it should be noted that they exhibit behavior similar to type 11 beams. For each of the beams, the highest strain values are concentrated on the beam section located near the point of load application, i.e., under concentrated forces, which confirms the previous statement that the peeling-off caused at the shear crack was the initiating cause of the strip debonding. This is also confirmed by the cracking pattern of the reinforced concrete beams shown in Figure 16—the crack concentration including shear cracks in the mentioned area. Slightly lower strain values of BW5 beams can be seen. At the level of 90% of the ultimate load, these values do not exceed 6‰ anywhere, while in the case of other types of beams, they reach values closer to 6.5‰. This may be due to the use of strip and anchorage overlap, which resulted in additional stiffening of the tested element and simultaneous reduction of maximum strains.

### 3.5. Crack Analysis

**Figure 18:**
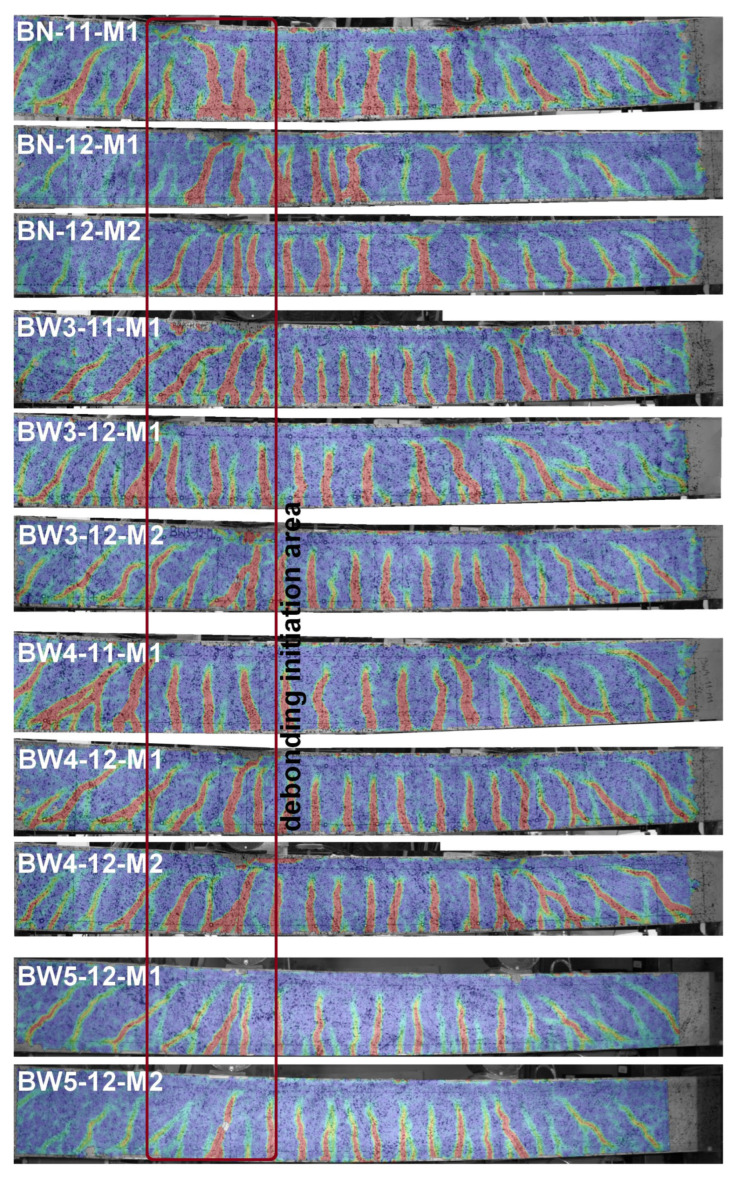
Comparison of RC beams with crack at 90% of the ultimate load.

**Figure 19:**
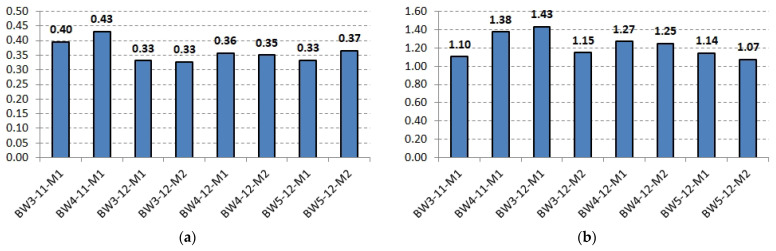
Measured crack widths in the recorded area before beam failure: (**a**) average values, (**b**) maximum values.

The crack analysis was based on the results obtained from the DIC system, including the image of the strains of the recorded surface (Figure 18) and the measurement of the crack width based on extensometers created at the height of the gravity of the tensile reinforcement at the place where the cracks occurred (Figure 19). Before the failure of the beams, a concentration of both perpendicular and diagonal cracks is visible under the actuators, mainly at the sliding support. Thus, the measured values of the crack width are the largest. Slightly smaller deformations are visible in the case of the BW5 beams, but this is mainly due to the use of a different version of the DIC system equipped with significantly higher-resolution sensors. Both the recorded video of the moment of beam failure and the obtained results confirm that the location marked in Figure 18 was the place where the CFRP strip debonding was initiated.

As for the cracking pattern, it is typical for this type of test—a four-point loading test, because the cracking is usually concentrated under the force and mainly on the sliding support side, which can also be observed in the case of unreinforced beams and the results presented in similar works [9,19,26].

The differences in the crack width values between individual beams are mainly due to the number of recorded cracks, i.e., with the increase in the number of cracks, their average width decreases.

## 4. Discussion

The main objective of the conducted research was to verify the effectiveness of the anchoring system proposed by the authors for the NSM CFRP strip implemented using a composite anchor made of CFRP fiber. Unfortunately, the obtained results are not fully satisfactory, because the obtained strengthening efficiency values, regardless of the anchoring used, are similar. The authors see two main reasons for this result. The first is a poor static scheme—too small spacing of the load application points. The place of the CFRP strip ending and the anchoring itself were made too far from the section of the constant moment, which in itself limited the possibility of debonding the strip ending. High strain values and consequently the crack widths located in the area under the load application caused the strip to peel off (vertical crack opening displacement). Strips adhesively bonded using the NSMR method are particularly susceptible to this type of destruction due to the limited possibility of adapting to local deformations due to the shape of their cross-section.

In all the tested beams, debonding occurred rapidly over practically the entire length of the strip. This releases the possibility of a plastic joint forming, which cannot be stopped by the applied anchoring, because the strain change over the entire section of debonding is no longer limited by anything. The use of anchoring and additional prestressing is able to delay the beam’s failure. In this case, local debonding of the strip without losing the anchorage will still limit the increase in strains of the beam’s tensile fibers. In the case of the tested beams, strain concentration occurred at the place of the largest crack, i.e., the place of debonding initiation under the point of force application, inevitably leading to the crushing of the compression zone. In the remaining length of the beam, strains decreased, and cracks were closed. Therefore, failure occurred despite the lack of debonding of the CFRP strip at the connection point with the fiber anchor.

It should be emphasized that in the case of CFRP strips used in the NSM method, the strip cross-section is higher and narrower than in the case of thin strips or sheets used in the EBR method. The greater moment of inertia of the strip and, consequently, greater stiffness makes this solution much more sensitive to the impact of vertical crack opening displacement in shear cracks. In this case, debonding will occur rapidly because the strip will be torn out of the groove, and at the same time, the horizontal crack opening displacement in shear cracks will contribute to a rapid increase in the section over which debonding occurs.

In subsequent studies, the authors plan to change the static scheme and the anchoring place. The initial load of the beams on the formation of cracks should also be taken into account, together with the strengthening of the beam under load. To fully reflect the actual working conditions of the structure, it is also necessary to supplement the research with cyclic loading.

As for the anchorage itself, it is necessary to test different configurations taking into account: different lengths of the anchor dowel/connection with the CFRP strip, different lengths of the anchor dowel, different cross-sections of the anchor, and the ratio between the cross-sectional area of the anchor and the CFRP strip. Based on the collected data, guidelines for the implementation and design of the proposed solution will be developed.

## 5. Conclusions

This manuscript describes the concept of anchoring NSM CFRP strips using an anchor made of fiber cord and the results of the load-deflection relationship and strain analyses of the unstrengthened and strengthened RC beams with and without additional anchoring. All beams were tested under a four-point static force-controlled load. The analysis was based on measurements made with the non-contact ARAMIS 5M and SRX digital image correlation (DIC) system. Three configurations of strengthened RC beams were compared (one without and two configurations with additional anchoring). The most important conclusions from the research conducted are presented below:

- As expected, all reinforced RC beams failed as a result of the debonding CFRP strip (practically along its entire length) at the CFRP strip interface—the top layer of CFRP strip fibers remained attached to the resin filling the groove after the beam failure. This destruction was initiated by strip peeling-off at a shear crack in the area under the load application point, due to vertical crack opening displacement (mainly on the sliding support side).
- Due to the debonding of the CFRP strip, the applied anchoring systems were unable to improve the load-bearing capacity of the beams compared to beams without additional anchoring.
- The lack of debonding in the strip at the connection section, the overlap with the CFRP anchor in the BW5-12-M1 beams, and the slightly higher stiffness of both BW5 RC beams can be considered a partial success.
- The value of strengthening efficiency was as follows: for the BW3 type beams strengthened with an NSM CFRP strip anchored at the ends (anchor fibers fan-folded on the beam surface at the strip end), it was 31–33%, while for the BW4 type beams strengthened only with an NSM CFRP strip without additional anchorage, it was 33–36%. In the case of BW5 beams, very divergent results were obtained, i.e., for the BW5-12-M2 beam the highest value of strengthening efficiency was obtained among all beams, equal to 37%, while for the BW5-12-M1 beam, the lowest was 29%.
- The relationship between force and mid-span deflection was practically the same for each of the strengthened RC beams.
- Analysis of the vertical displacements for the sections along the beam at the height of the center of gravity of the tension reinforcement indicates that each of the strengthened beams experiences a vertical crack opening displacement in shear crack forming under the point of force application near the sliding support.
- In the same area, before the failure of the beams, a concentration of both perpendicular and diagonal cracks was visible. This is the location where cracks with the maximum width among those recorded on the surface of the tested beams by the DIC system occurred.

When designing beam strengthening, special attention should be paid to the locations of concentrated forces and shear cracks, because even additional strip anchorage is not able to fully protect against debonding caused by horizontal and vertical crack opening displacement in a shear crack, which was the case in the described tests. Failure of this type is known but unfortunately insufficiently investigated, mainly in the case of NSM reinforcements, and an appropriate bond model needs to be developed.

## Figures and Tables

**Table 1:** Materials and specimen parameters.

Specimen	Concrete		Steel Bars			CFRP Strip				CFRP Anchor		
	f_c_ ^1^ [MPa]	E_c_ ^2^ [GPa]	A_s1_ ^3^ [cm^2^]	f_y_ ^4^ [MPa]	E_s_ ^5^ [GPa]	Typ ^6^	A_f_ ^7^ [mm^2^]	f_f_ ^8^ [MPa]	E_f_ ^9^ [GPa]	A_f,A_ ^10^ [mm^2^]	f_f,A_ ^11^ [MPa]	E_f,A_ ^12^ [GPa]
BN-11-M1	50.1	35.7	3.08	505.8	219.8	-	-	-	-	-	-	-
BN-12-M1	57.1	37.1	3.08	505.8	219.8	-	-	-	-	-	-	-
BN-12-M2	57.1	37.1	3.08	505.8	219.8	-	-	-	-	-	-	-
BW3-11-M1	50.1	35.7	3.08	505.8	219.8	II	37.5	3100	170	28	2000	230
BW3-12-M1	57.1	37.1	3.08	505.8	219.8	II	37.5	3100	170	28	2000	230
BW3-12-M2	57.1	37.1	3.08	505.8	219.8	II	37.5	3100	170	28	2000	230
BW4-11-M1	50.1	35.7	3.08	505.8	219.8	I	37.5	3100	170	-	-	-
BW4-12-M1	57.1	37.1	3.08	505.8	219.8	I	37.5	3100	170	-	-	-
BW4-12-M2	57.1	37.1	3.08	505.8	219.8	I	37.5	3100	170	-	-	-
BW5-12-M1	53.5	36.4	3.08	505.8	219.8	III	37.5	3100	170	28	2000	230
BW5-12-M2	53.5	36.4	3.08	505.8	219.8	III	37.5	3100	170	28	2000	230

^1^ f_c_—compressive strength of concrete (cylinder compressive strength); ^2^ E_c_—modulus of elasticity of concrete; ^3^ A_s1_—cross-sectional area of reinforcement in the tension zone: 2Ø14; ^4^ f_y_—yield strength of reinforcement; ^5^ E_s_—modulus of elasticity of steel; ^6^ Type (I, II, III)—determines the method of reinforcing the reinforced concrete beam; ^7^ A_f_—cross-sectional area of FRP: Sika CarboDur S NSM 1.525 CFRP strip 37.5 mm^2^; ^8^ f_f_—tensile strength of the FRP; ^9^ E_f_—modulus of elasticity of FRP; ^10^ A_f,A_—cross-sectional area of FRP: SikaWrap^®^ FX-50 CFRP anchor ~28 mm^2^; ^11^ f_f,A_—tensile strength of the FRP; ^12^ E_f,A_—modulus of elasticity of FRP.

**Table 2:** Failure modes and beam load-bearing capacities.

Specimen	Expected Ultimate Load [kN]	Ultimate Load [kN]	*η_f_* * [%]	Failure Mode
BN-11-M1	-	42.8	-	-
BN-12-M1	-	44.0	-	-
BN-12-M2	-	43.8	-	-
BW3-11-M1	59.2	56.9	33	Debonding of the CFRP strip at the left end and middle of the FRP material followed by compressive concrete crushing on the top surface of the beam.
BW3-12-M1	59.7	57.9	32	Debonding of the CFRP strip along its entire length followed by concrete compressive crushing on the top surface of the beam.
BW3-12-M2		57.5	31	
BW4-11-M1	59.2	58.4	36	Debonding of the CFRP strip along its entire length followed by concrete compressive crushing on the top surface of the beam.
BW4-12-M1	59.7	58.6	33	
BW4-12-M2		59.1	35	
BW5-12-M1	59.5	56.6	29	Debonding of the CFRP strip followed by compressive concrete crushing on the top surface of the beam. No debonding at the anchor point and the strip and fiber anchor overlap.
BW5-12-M2	59.5	60.2	37	Debonding of the CFRP strip along its entire length followed by concrete compressive crushing on the top surface of the beam.

* strengthening efficiency *η_f_* = (*F_u_* − *F*_0_)/*F*_0_, where *F_u_*—ultimate force for the strengthened beam, *F*_0_—ultimate force for the reference beam (unstrengthened).

## Data Availability

The original contributions presented in this study are included in the article. Further inquiries can be directed to the corresponding author.
